# Suramin enhances the urinary excretion of VEGF-A in normoglycemic and streptozotocin-induced diabetic rats

**DOI:** 10.1007/s43440-021-00236-0

**Published:** 2021-02-26

**Authors:** Gabriela Chyła, Kornelia Sałaga-Zaleska, Kamil Dąbkowski, Agnieszka Kuchta, Maciej Jankowski

**Affiliations:** Department of Clinical Chemistry, Medical University of Gdańsk, Dębinki 7, 80-210 Gdańsk, Poland

**Keywords:** Diabetes, Glomerulus, Kidney, P2-receptors, Streptozotocin, Vascular endothelial growth factor A

## Abstract

**Background:**

Vascular endothelial growth factor A (VEGF-A) and P2-receptors (P2Rs) are involved in the pathogenesis of diabetic nephropathy. The processing of VEGF-A by matrix metalloproteinases (MMP) regulates its bioavailability. Since the ATP-induced release of MMP-9 is mediated by P2Rs, we investigated the effect of suramin on VEGF-A excretion in urine and the urinary activity of total MMP and MMP-9.

**Methods:**

The effect of suramin (10 mg/kg, *ip*) on VEGF-A concentration in serum and its excretion in urine was investigated in streptozotocin (STZ)-induced diabetic rats over a 21-day period. The rats received suramin 7 and 14 days after a single STZ injection (65 mg/kg, *ip*). A 24-h collection of urine was performed on the day preceding the administration of STZ and the first administration of suramin and on the day before the end of the experiment. The VEGF-A in serum and urine, albumin in urine, and total activity of MMP and MMP-9 in urine were measured using immunoassays.

**Results:**

Diabetic rats are characterized by a sixfold higher urinary excretion of VEGF-A. Suramin potentiates VEGF-A urinary excretion by 36% (*p* = 0.046) in non-diabetic and by 75% (*p* = 0.0322) in diabetic rats but it did not affect VEGF-A concentration in the serum of non-diabetic and diabetic rats. Urinary albumin excretion as well as total MMP and MMP-9 activity was increased in diabetic rats, but these parameters were not affected by suramin.

**Conclusion:**

Suramin increases the urinary excretion of VEGF-A in normoglycemia and hyperglycaemia, possibly without the involvement of MMP-9. Suramin may be used as a pharmacological tool enhancing VEGF-A urinary secretion.

## Introduction

Diabetic nephropathy is a major complication of diabetes leading to end-stage renal disease and is currently a major cause of morbidity and mortality in diabetic patients. It is characterized, at the organ level, by progressive kidney damage reflected by an increase in albumin excretion in urine and a decline in glomerular filtration. Moreover, at a cellular level, it is characterized by the dysfunctions of vascular endothelial cells and glomerular visceral epithelial cells called podocytes [1]. These conditions may be related to changes in membrane receptor expression/activity and the dysregulation of angiogenic factors [2, 3]. Since glomerular endothelial cells and podocytes cross-talk, a disturbance occurring in one of them may be transmitted to the other, thus aggravating and accelerating the damage to the glomerulus [4]. Among the receptors whose expression/activity is altered are those activated by the extracellular nucleotides called P2-receptors (P2Rs). One of the P2Rs that may be relevant is the P2X7 receptor (P2X7R). For example, the increased glomerular expression of the ATP-sensitive P2X7R in a diabetic rat model has been shown [5], and we have previously shown that glomerular microvasculature reactivity to an agonist of P2X7R is increased in streptozotocin (STZ)-induced diabetes [6]. While it has been shown that P2Rs are involved in intraglomerular extracellular matrix protein accumulation in diabetic glomeruli [7], diabetic nephropathy is associated with both systemic and local renal inflammation with the participation of crucial inflammatory cells expressing P2Rs influencing the release of cytokines, which further act via the nuclear transcription of factor-kappa B [8]. On the other hand, P2Rs influence the activity of the matrix metalloproteinases (MMPs), key enzymes in the extracellular matrix metabolism, as shown by studies in which the ATP-induced rapid release of matrix metalloproteinase-9 (MMP-9) is mediated by P2X7R [9]. MMP-9 expression in human podocytes and enhanced MMP-9 urinary concentrations in patients with diabetic nephropathy have also been reported [10]. In turn, MMPs regulate the bioavailability of the vascular endothelial growth factors family (VEGF), major angiogenesis and vascular permeability factors [11]. VEGF members, among which VEGF-A plays a key role, facilitate cellular responses by binding to tyrosine kinase receptors on a cell’s surface [11]. VEGF-A is produced in glomeruli by podocytes and diffuses towards capillary lumens, where it reaches the glomerular endothelial cells and causes an increase in glomerular permeability to water [12, 13]. The genetic-based study has provided evidence that increased local renal VEGF levels affect the glomerular endothelium fenestration, which is a surrogate marker for local VEGF-A bioactivity [14]. Importantly, elevated VEGF-A levels are associated with glomerular pathologies in diabetic nephropathy [15]. On the other hand, the results of the genetic-based studies suggest that VEGF-A in diabetic kidneys may play protective role [16].

Thus, we hypothesized that VEGF-A bioavailability in glomeruli may be regulated by P2Rs. To reach the study’s aim, non-toxic suramin, a broad-spectrum P2R antagonist, was used in STZ-induced diabetic rats, and the urinary excretion of VEGF-A and activities of MMP and MMP-9 in urine were measured.

## Materials and methods

### Ethical approval

The experiments were conducted in accordance with the European Convention for the Protection of Vertebrate Animals Used for Experimental and Other Scientific Purposes and approved by the local Bioethics Commission in Bydgoszcz, Poland (Approval no 35/2017).

### Animals

The studies were performed on male Wistar rats (Tri-City Academic Laboratory Animal Centre, Gdańsk, Poland), weighing 200–250 g, aged 8–10 weeks, housed under a 12-h light/12-h dark cycle, and fed a standard pellet diet (Labofeed B, Kcynia, Poland) and water ad libitum. The rats were divided into four groups (*n* = 7 for each group):

1. control, citrate buffer injected at day + 1, CON;
2. control + suramin (10 mg/kg b.w., *ip*), injected at days + 7 and + 14 after the citrate buffer injection, SUR;
3. streptozotocin (65 mg/kg b.w., *ip*), injected at day + 1, STZ;
4. streptozotocin + suramin (10 mg/kg b.w., *ip*), injected at days + 7 and + 14 after STZ injection, STZ + SUR.

**Fig. 1:**
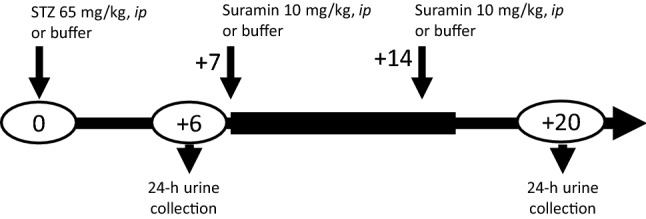
Scheme of the experimental procedure

The experiments were performed on rats with tail blood glucose concentrations greater than 11.1 mmol/l measured at day + 6 after STZ injection. The effectiveness of hyperglycaemia induction was 87.5%. Twenty-four-hour urine samples were collected in metabolic cages (Tecniplast, Italy) at days + 6 and + 20 after the STZ injection. The urine was collected in tubes containing protease inhibitors (5 × 10^−4^ M PMSF, 10^−6^ M leupeptin) and 3 × 10^−3^ M NaN_3_. At the end of the experiment on day + 21, all animals were overdosed with anaesthesia, their thoraxes were opened and blood was drawn by cardiac puncture to preserve the serum of each rat, which resulted in the death of the rats. A schematic of the procedure is depicted in Fig. 1.

### Analytic methods

Blood glucose was determined with an Accu-Chek™ Performa glucometer (Roche, Basel, Switzerland), and urine volume was determined gravimetrically. Immunoenzymatic assays were used to measure the concentration of rat albumin (AssayPro, USA, Cat. No. ERA3201-1) and rat VEGF-A (Thermo Scientific, USA, Cat. No. ERVEGFA). A fluorometric assay was used to measure the activity of total MMP (AnaSpec, USA Cat. No. AS-71158) and MMP-9 (AnaSpec, USA Cat. No. AS-71155), while the creatinine concentration was measured by the enzymatic method (Wiener lab., Argentina).

### Statistical analysis

The statistical analyses were performed using Statistica 13.3 (TIBCO Software). A Shapiro–Wilk test was used to test the determined normality of the distribution of variables; continuous variables were expressed as mean ± SE (standard error). Statistical significance between the groups was determined using two-way ANOVA and post hoc Tukey's multiple comparisons. A paired *t* test was used to assess changes in repeated measures. Univariate correlations were assessed using standardized Pearson coefficients. Differences were considered significant for *p* < 0.05.

### Materials

Leupeptin was purchased from Merck KGaA (Darmstadt, Germany). All other agents were purchased from Avantor™ Performance Material Poland S.A. (Gliwice, Poland).

## Results

**Fig. 2:**
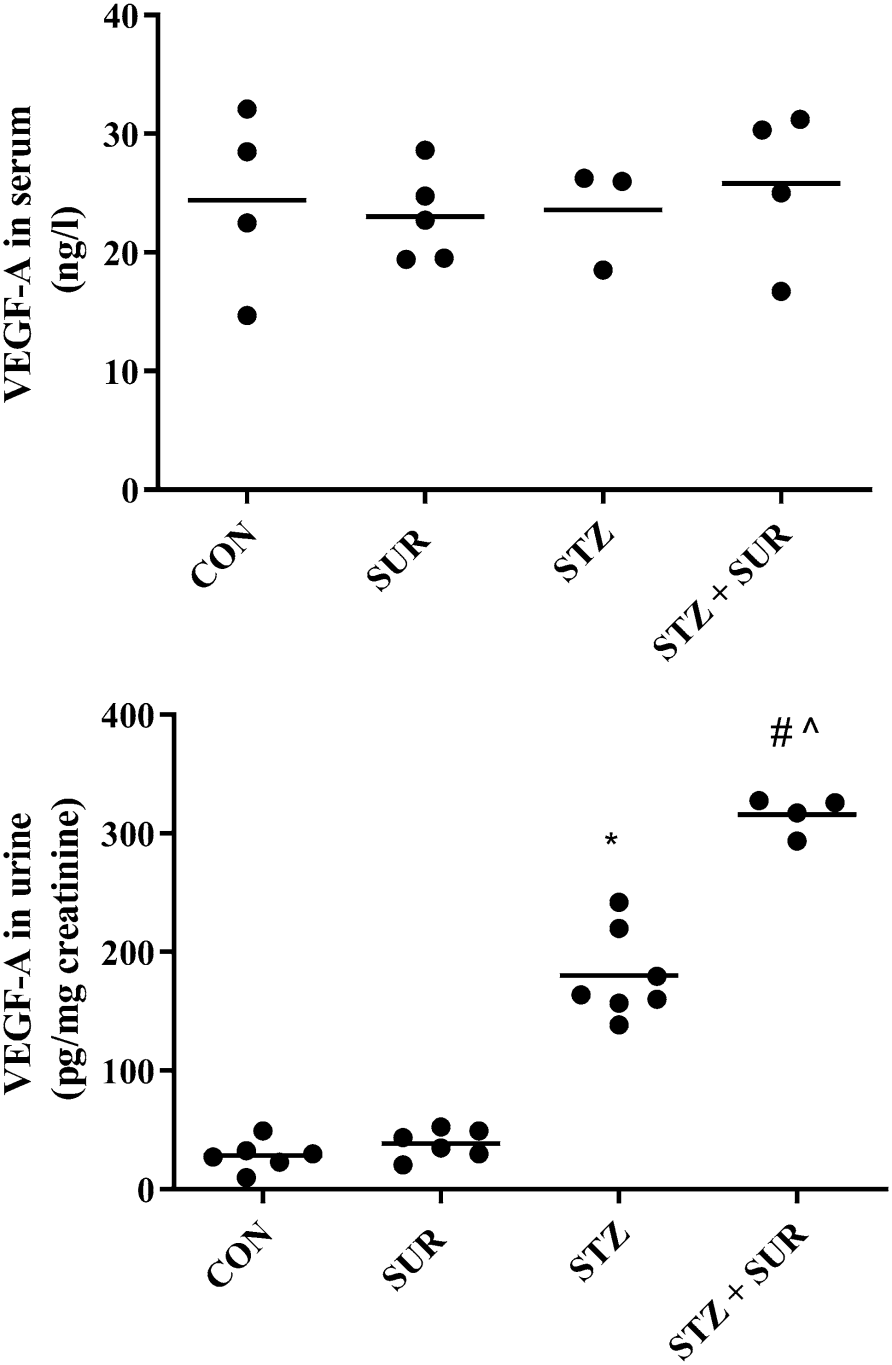
The effects of suramin (10 mg/kg, *ip*) on VEGF-A concentration in serum (**a**) and urinary excretion of VEGF-A (**b**) in non-diabetic and streptozotocin-induced (65 mg/kg, *ip*) diabetic rats. Non-diabetic and 1-week diabetic rats were injected with PBS (CON and STZ) and suramin (SUR and STZ + SUR) once per week for 2 weeks. The results are presented as individual data points with means. Statistical significance was determined using two-way ANOVA with a Tukey post hoc test, *p < 0.0001 vs. CON, ^#^*p* < 0.0001 vs. SUR, ^*p* < 0.0001 vs. STZ.

**Fig. 3:**
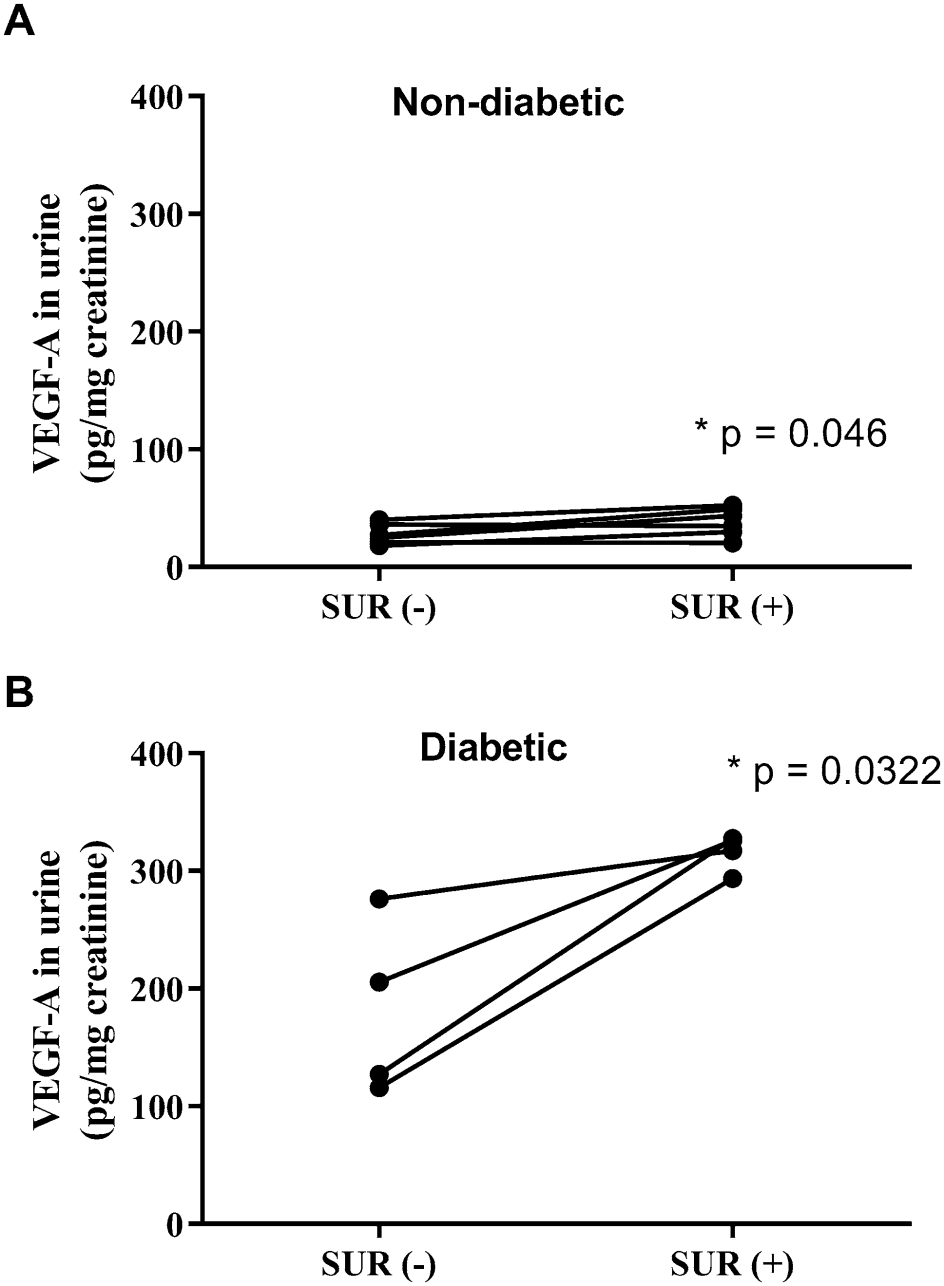
The effects of suramin (10 mg/kg, *ip*) on VEGF-A urinary excretion in non-diabetic and streptozotocin-induced (65 mg/kg, *ip*) diabetic rats. The results are presented as individual data points before, SUR (−), and after 14 days of suramin exposition, SUR (+), in non-diabetic (**a**) and diabetic (**b**) rats. Statistical significance was determined using a paired samples *t* test, *p as indicated

The serum concentrations of VEGF-A were CON, 24.4 ± 3.8 ng/l; SUR, 23.0 ± 1.7 ng/l; STZ, 23.6 ± 2.5 ng/l; and STZ + SUR: 25.8 ± 3.3 ng/l (Fig. 2a). The main effects of diabetes (F_1,12_ = 0.1113, *p* = 0.744) and suramin (F_1,12_ = 0.0187, *p* = 0.893) on VEGF-A concentration in serum were not significant. Figure 2b presents results of VEGF-A excretion in urine. Two-way ANOVA revealed the significant main effects of diabetes (F_1,19_ = 453.25, *p* < 0.0001), suramin (F_1,19_ = 52.277, *p* < 0.0001), and a diabetes-by-suramin interaction (F_1,19_ = 39.095, *p* < 0.0001). Post hoc comparisons showed a sixfold increased VEGF-A excretion in diabetic rats compared with non-diabetic rats (180 ± 14 pg/mg creatinine vs. 29 ± 5 pg/mg creatinine, *p* < 0.0001). It is noteworthy that suramin additionally increases the urinary excretion of VEGF-A by 76% in diabetic rats (180 ± 14 pg/mg creatinine vs. 316 ± 8 pg/mg creatinine, *p* < 0.0001). An intergroup data analysis of VEGF-A excretion in urine under the influence of suramin did not reveal a statistically significant effect of suramin in non-diabetic rats. However, as shown in Fig. 3a, b an intragroup analysis of urinary VEGF-A excretion using a paired samples *t* test shows that 2 weeks exposure to suramin leads to a statistically significant increase in urinary VEGF-A excretion of 36% in non-diabetic rats (28 ± 4 pg/mg creatinine vs. 38 ± 5 pg/mg creatinine, *p* = 0.046) (Fig. 3a) and of 75% in diabetic rats (181 ± 37 pg/mg creatinine vs. 316 ± 8 pg/mg creatinine, *p* = 0.0322) (Fig. 3b).

**Fig. 4:**
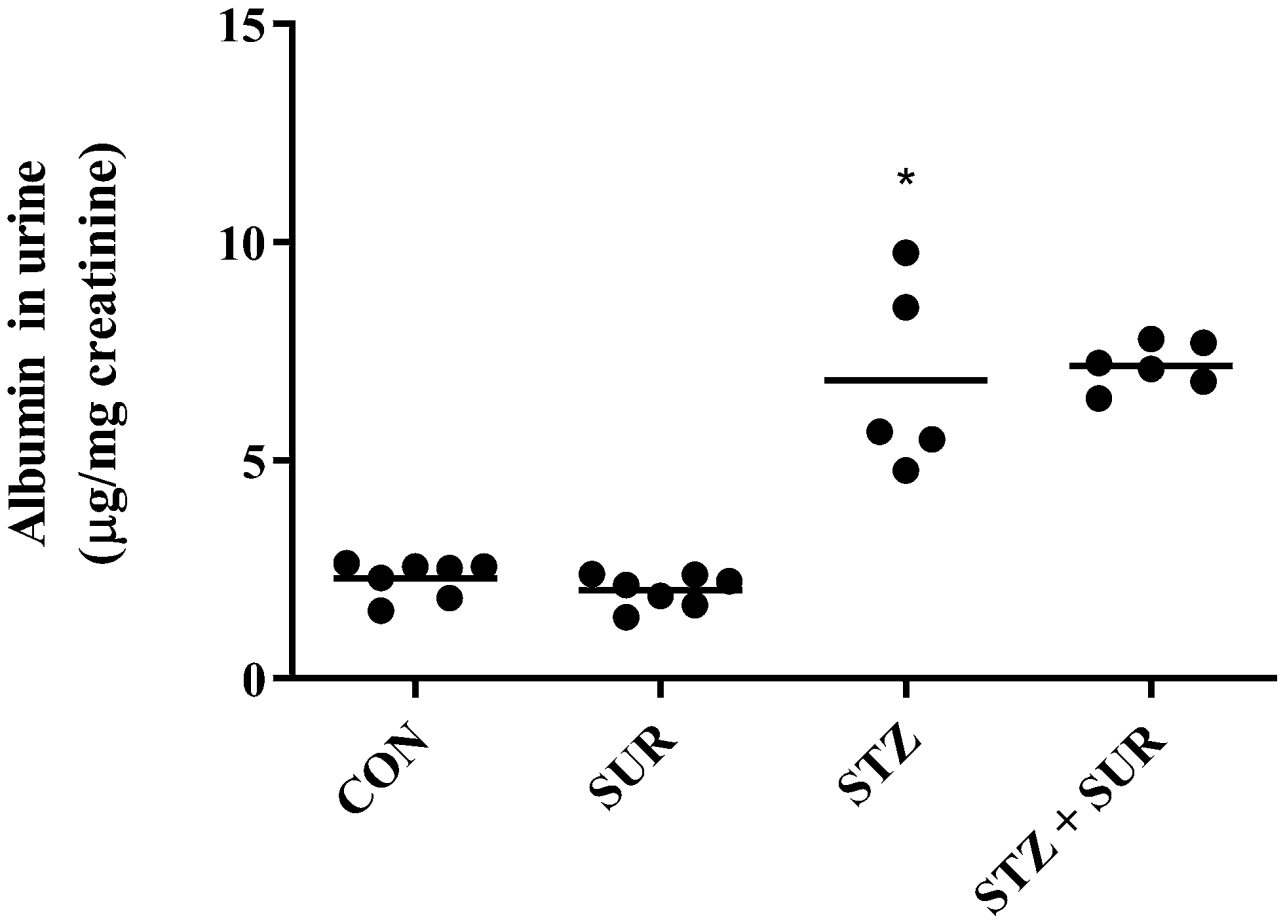
The effect of suramin (10 mg/kg, *ip*) on urinary albumin excretion in non-diabetic and streptozotocin-induced (65 mg/kg, *ip*) diabetic rats. Non-diabetic and 1-week diabetic rats were injected with PBS (CON and STZ) and suramin (SUR and STZ + SUR) once per week for 2 weeks. The results are presented as individual data points with means. Statistical significance was determined using two-way ANOVA with Tukey post hoc test, *p < 0.0001 vs. CON, ^#^*p* < 0.0001 vs. SUR

Figure 4 shows the results of urinary albumin excretion. Two-way ANOVA revealed a significant main effect of diabetes (F_1,21_ = 137.2, *p* < 0.0001). No significant main effect of suramin or interaction was present. The albumin excretion in diabetic rats was threefold higher in diabetic rats compared with non-diabetic rats (6.8 ± 1.0 µg/mg creatinine vs. 2.3 ± 0.2 µg/mg creatinine, *p* < 0.0001). Of note, there was significant correlation between urinary excretion of albumin and VEGF-A in diabetic rats (*r* = 0.8817, *p* < 0.048).

**Fig. 5:**
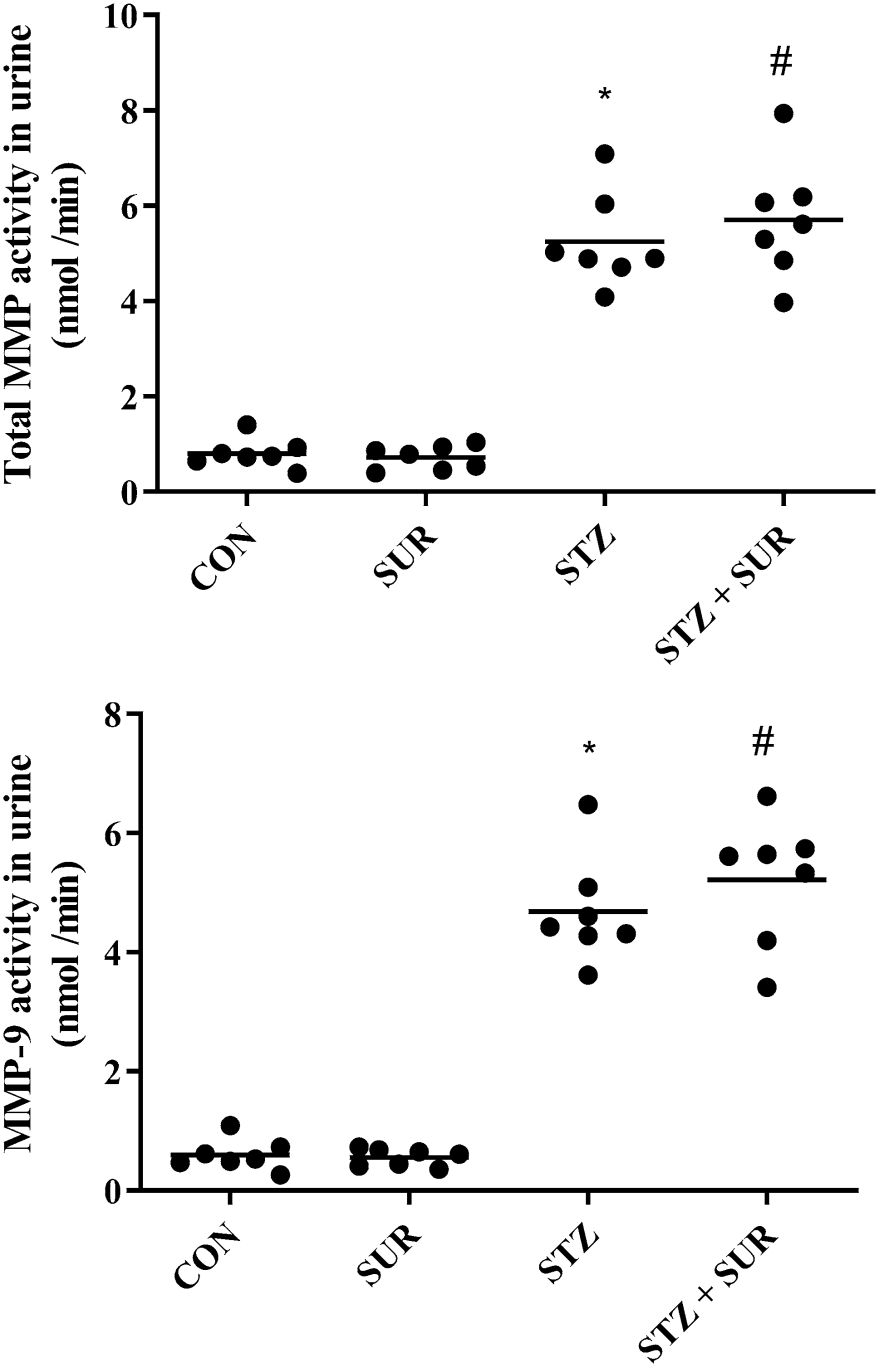
The effects of suramin (10 mg/kg, *ip*) on urinary activities of total matrix metalloproteinases (MMP) (A) and matrix metalloproteinase-9 (MMP-9) (B) in non-diabetic and streptozotocin-induced (65 mg/kg, *ip*) diabetic rats. Non-diabetic and one-week diabetic rats were injected with PBS (CON and STZ) and suramin (SUR and STZ + SUR) once per week for 2 weeks. The results are presented as individual data points with means. Statistical significance was determined using two-way ANOVA with a Tukey post hoc test, *p < 0.0001 vs. CON, ^#^*p* < 0.0001 vs. SUR

The results of total MMP and MMP-9 activities in urine are presented in Fig. 5a, b. Two-way ANOVA revealed the significant main effects of diabetes on total MMP (F_1,24_ = 231.0, *p* < 0.0001) and MMP-9 activities (F_1,24_ = 261.3, *p* < 0.0001). Diabetic rats were characterized by a 6.5-fold higher activity of total MMP (5.25 ± 0.38 nmol/min vs. 0.81 ± 0.12 nmol/min, *p* < 0.0001, Fig. 5a) and about an eightfold higher activity of MMP-9 (4.69 ± 0.34 nmol/min vs. 0.59 ± 0.09 nmol/min, Fig. 5b) than non-diabetic rats.

The main effects of suramin on total MMP (F_1,24_ = 0.3476, *p* = 0.561) and MMP-9 activities (F_1,24_ = 0.8143, *p* = 0.3758) were not significant, nor were the diabetes-by-suramin interactions.

## Discussion

The present study has provided evidence that the urinary excretion of VEGF-A may be pharmacologically modified by suramin. Polysulfonated naphthylurea is used in laboratories as a broad-spectrum antagonist of P2Rs and in clinics for a wide array of potential applications, from parasitic and viral diseases to cancer, snakebite and autism [17]. In our experiments, the administration of suramin (10 mg/kg, ip) once per week for 2 weeks leads to a significant enhancement of the urinary excretion of VEGF-A, both in normoglycemic (about 36%) and STZ-induced hyperglycaemic rats (about 60–70%). This finding seems to be of considerable importance for understanding the pathogenesis of diabetic nephropathy and perhaps extending the pharmacological possibilities of kidney protection in diabetic patients. VEGF-A is a key secreted glycoprotein of the VEGF family of heparin-binding growth factors that play an important role in the regulation of glomerular structure and function and may also influence the outcome of diabetic kidney disease. The upregulation of VEGF-A in glomeruli is observed in the early stages of diabetes [15], and on this observation anti-VEGF-A therapy is based. In STZ-induced diabetic rats, treatment with monoclonal anti-VEGF antibodies decreased hyperfiltration, albuminuria and glomerular hypertrophy [18]. However, the outcomes of VEGF-A inhibition in experimental diabetes have been conflicting [19]. Regardless, there is strong evidence that VEGF-A plays a pivotal protective role in the pathogenesis of microangiopathic processes [20]. Moreover, the results of the genetic-based studies have provided evidence that the upregulation of VEGF-A in diabetic kidneys protects the microvasculature from injury [16].

To achieve significant therapeutic benefits, while at the same time taking into account the short half-life and high susceptibility to degradation of VEGF-A in vivo, therapeutic management may require intrarenal administration, which in clinical conditions seems unlikely to be achieved today. Thus, the therapeutic challenge is to find the agents that affect the renal/intraglomerular concentration of VEGF-A that could be administered to patients on an outpatient basis.

Suramin as reported is mostly accumulated in the kidneys and half-life allows it to be administered once per week [21]. It has been previously shown that suramin administered intraperitoneally twice at weekly intervals prevented the rise of 24-h proteinuria and attenuated renal fibrosis and glomerular damage in a remnant kidney model of chronic kidney disease [22]. In our experiment model of early stage diabetes, we have noticed not only significant changes in the urinary excretion of albumin in diabetic rats but also in normoglycemic rats, both treated with suramin. Our observation is supported by results obtained from db/db mice in which delayed administration of a single dose of suramin did not affect protein excretion in 9- and 17-week mice, suggesting that suramin did not affect the number of podocytes or podocyte-specific proteins involved in the pathogenesis of albuminuria in this mice strain [23].

As VEGF-A is mainly produced in podocytes, the increased urinary excretion of VEGF-A indicates increased production of this cytokine in podocytes, from where it passes through the glomerular filter into the lumen of capillaries and interacts there with its receptors located on glomerular endothelial cells. Using a model of VEGF transport against glomerular filtration flow, it has been calculated that about one-third of podocyte-derived VEGF-A reaches the glomerular endothelial cells via diffusion [13]. Moreover, the recent study has provided evidence of unexpected positive correlation between single-nephron glomerular filtration rate with VEGF-A back diffusion [14]. Taking into account that suramin enhances the urinary excretion of VEGF-A by 60–70% in diabetic rats, one may expect that local concentration in the glomerulus may by increased by about 20%. We measured the VEGF-A concentration in blood and did not find significant differences in this parameter between experimental groups. This suggests a possible effect of suramin on local intrarenal rather than systemic VEGF-A concentrations, which is consistent with the pharmacokinetic properties of suramin accumulation in the kidneys. One should also take into account the results of experiments carried out on cultured endothelial cells, the results of which may suggest that suramin may act as a factor limiting the process of angiogenesis stimulated by VEGF-A [24].

The mechanism of how suramin induces the elevation of VEGF-A excretion in urine is open to question. The bioavailability of VEGF-A is regulated by its processing by MMPs, especially MMP-9, expressed in podocytes and regulated by P2X7R upregulated in diabetes [5, 9, 25]. We observed increased activities of MMP and MMP-9 in diabetic rats, which are consistent with clinical studies in which, for example, the level of urinary MMP-9 is increased in patients with diabetic nephropathy, and positively correlates with the clinical stage of the disease [10]. The increase in MMP-9 activity may be responsible for diabetes-associated enhanced renal production of VEGF-A [10]. In our experiments, the urinary activities of MMP and MMP-9, both in normoglycemic and hyperglycaemic rats, were not affected by suramin. The pharmacology data for P2Rs suggest that the direct in vivo action of suramin on MMPs via P2X7R is hardly possible because of low potency, namely IC_50_ ~ 70 µM at human P2X7R receptors and IC_50_ > 300 µM at rat P2X7R receptors [26, 27]. It seems possible that the suramin concentration in the blood of our experimental rats may not reach adequate levels to block P2X7Rs because it has been shown that the administration of suramin at a dose of 10 mg/kg twice per week yields plasma concentrations below 50 µM [28].

In conclusion, our results show that suramin administered once per week enhanced already increased VEGF-A excretion in diabetic rats and is also able to increase the physiological level of VEGF-A excretion in normoglycemic rats. We believe that our observations may contribute to the extension of therapeutic possibilities in diabetic patients aimed at the protection of glomerular microcirculation. This, in turn, will result in slowing down the pathological processes underlying the development of diabetic kidneys.
